# Using mHealth to promote parents’ brushing of preschool children’s teeth: a protocol for a randomized factorial trial using the Multi-phase Optimization Strategy (MOST)

**DOI:** 10.1186/s13063-021-05931-0

**Published:** 2022-01-06

**Authors:** Merna Ihab, Wafaa Essam El Din, Nour Ammar, Randa Yassin, Maha El Tantawi

**Affiliations:** Department of Pediatric Dentistry and Dental Public Health, Faculty of Dentistry, Alexandria University, Champollion St., Azarita, Alexandria, 21527 Egypt

**Keywords:** Multi-phase optimization strategy, MOST, Optimization trial, Preparation phase, Oral hygiene, Motivational interviewing, mHealth, Preschoolers, Early childhood caries

## Abstract

**Background:**

Early childhood caries is a highly prevalent disease affecting young children. Parental brushing of children’s teeth is recommended during preschool years. Interventions to promote parental brushing of children’s teeth are assessed as a package in randomized clinical trials and the efficacy of separate components is not known.

**Methods and analysis:**

The aim of this study is to develop an optimized behavior modification intervention to increase parents’ brushing of their pre-school children’s teeth using the multi-phase optimization strategy (MOST) guided by the Theory of Planned Behavior. Behavior change will be assessed by the percent reduction in children’s dental plaque index after 6 months and parents reporting of toothbrushing frequency. Two phases of MOST will be carried out. First, the preparation phase comprises the development of a conceptual framework, identifying candidate components, conducting a feasibility pilot study to assess the acceptability and the design features of three intervention components (motivational interviewing (MI), and two mobile health (mHealth) components: oral health promotion messages and storytelling videos delivered using WhatsApp messenger) in addition to setting an optimization objective. Second, the optimization phase constitutes a factorial trial assessing the three intervention components and developing the intervention by selecting the most effective components within the optimization constraint. Each component will be set at two levels: yes (the intervention is applied) and no (the intervention is not applied). A linear regression model will be used to assess the effect of the intervention components on the percent reduction in dental plaque index (primary outcome measure). The secondary outcome measure is the change in the frequency of parents’ brushing of the child’s teeth. The combination of components making up the new optimized intervention will be selected.

**Discussion:**

This will be the first study to apply the MOST framework in the field of dentistry. The results of this study can guide the development of an optimized behavior modification interventions using mHealth and MI.

**Trial registration:**

ClinicalTrials.gov, NCT04923581, Registered 11 June 2021.

**Supplementary Information:**

The online version contains supplementary material available at 10.1186/s13063-021-05931-0.

## Background

Early childhood caries (ECC) is the most prevalent chronic disease affecting children worldwide with high prevalence in Egypt exceeding 50% [1, 2]. ECC has adverse effects on the growth and development of children and their quality of life as well as that of their caregivers [3]. Regular toothbrushing with fluoridated toothpaste is a highly effective home care measure [4], which renders dental health education of caregivers one of the best approaches to control this disease [5].

Parents are responsible for brushing their children’s teeth at young age to prevent dental caries [6, 7]. The American Academy of Pediatric Dentistry recommends parental supervision of children’s toothbrushing during preschool years [8]. Barriers to parents’ brushing of their children’s teeth include inadequately supportive and organized home environment, absence of flexibility in daily activities, and parenting styles lacking positive reinforcement and involvement [9, 10]. In addition, the child’s development, level of co-operation, and the surrounding community-level factors may influence toothbrushing [10].

Several theories were proposed to explain health behaviors and how they are affected by various factors and barriers. These theories include the social cognitive theory, the theory of diffusion of innovation, the theory of planned behavior (TPB) [11], and the health belief model [12]. The TBP was used to decipher barriers to behavior change in oral health education programs [13], to design behavior change interventions [14], to improve interventions by assessing participants’ background [15], and to promote parental brushing of children’s teeth [16]. The TPB can guide the development of interventions to promote parents’ brushing of their children’s teeth by addressing the impact of barriers affecting parents’ perceived control, their attitude toward oral health, and the norms they perceive regarding the value of oral hygiene.

Several interventions were developed [17] to promote parents’ brushing of children’s teeth and were assessed using randomized clinical trials (RCTs). These interventions usually consist of multiple components to maximize the efficacy of the intervention and are evaluated as a single package [18]. The multi-phase optimization strategy (MOST) assesses the effectiveness of each component in the intervention separately. MOST is an engineering-inspired framework that focuses on developing and optimizing an intervention according to a specific optimization objective so that components of the interventions can later be evaluated for effectiveness as a package in an RCT [18]. The aim is not only to develop an effective intervention but an efficient one too. Understanding the mechanisms by which an intervention produces its effects makes it possible to build upon previous interventions in a more systematic and gradual means [18]. MOST consists of three phases: preparation, optimization, and evaluation phases. The preparation phase lays the groundwork for the development of the conceptual framework and the identification of candidate intervention components using a feasibility pilot study. In the optimization phase, the optimized intervention is constructed using optimization trials. Finally, the effectiveness of the optimized intervention can be confirmed in the evaluation phase through an RCT.

We aim to use the MOST framework to engineer a behavior modification intervention to promote parents’ brushing of their pre-school children’s teeth using fluoridated toothpaste and to select intervention components and component levels with the greatest efficacy within a predefined optimization objective. We will select intervention components based on the constructs of the TPB then test them for acceptability in a feasibility pilot study representing the preparation phase of MOST. This will be followed by an optimization factorial design trial to compare the selected components and select the components with the greatest efficacy within predefined optimization objectives.

## Methods/design

### Overview of the study

I- The preparation phase, including:

- Developing a conceptual framework to guide the design of the intervention
- Identifying candidate intervention components
- Conducting a feasibility pilot study to determine the acceptability of the intervention components and identify the preferred frequency and timing of providing them
- Setting an optimization objective

II- The optimization phase, including:

- Assessing the individual components using a factorial trial.
- Identifying the intervention components with greatest efficacy with the specified optimization objective.

#### I. Preparation phase

##### a. Conceptual framework

**Fig. 1:**
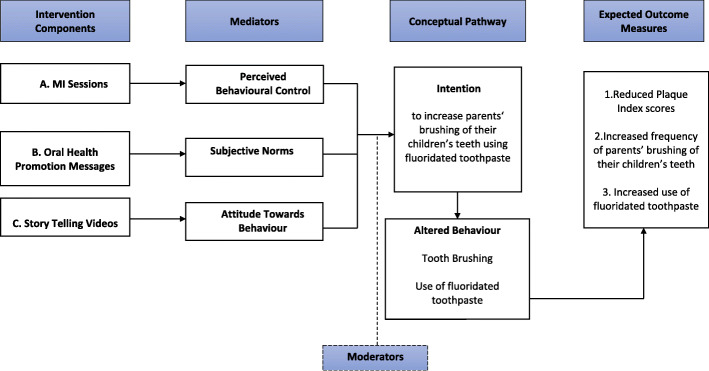
Conceptual framework based on the TPB

The TPB posits that intentions are predictors of behaviors and are affected by attitudes, subjective norms, and perceived control [19]. Individuals adopt beliefs about the outcomes of a behavior, and these beliefs contribute to their attitude. Subjective norms are the perception about the expectations of important others, and these contribute to the perception of social pressure and motivation to comply. Perceived control is divided into two components: self-efficacy which is the sense of ease/ difficulty in engaging in the behavior and controllability which is the extent to which the performance is up to the person. Parental attitude, self-efficacy, and intention are modifiable social-cognitive constructs that were reported to be significantly associated with preschool children’s oral hygiene behavior and can be targeted by behavior modification interventions [20]. In the proposed study, each TPB construct will be targeted by a component, and this is assumed to lead to the desired behavior modification (Fig. 1).

##### b. Identifying candidate components (Table 1)

Three components are proposed in addition to a core/constant component provided to all participants. The three components are motivational interviewing (MI), oral health promotion messages (OHPM), and storytelling (ST). In a previous study, MI outperformed traditional oral health education in improving patient behaviors and oral health perceptions and enhancing clinical indicators (e.g., plaque index, gingival index, bleeding on probing) [21]. Another study [22] reported that messages based on subjective norms were the most effective in inducing behavioral changes. The use of story-based approaches also showed promising results in improving children’s oral hygiene [23].

**Table 1:** Candidate intervention components

Intervention component	Target construct	Frequency of delivering the component	Method of delivery and setting
**Constant component**	Core knowledge	Once at baseline	Pamphlets in clinic
**MI**	Perception of control	1 MI session + 3 phone calls	Face to face in clinic + phone calls
**OHPM**	Perceived norms	Determined during the feasibility pilot study	mHealth: electronic messages via WhatsApp messenger
**ST videos**	Attitude	Determined during the feasibility pilot study	mHealth: electronic messages via WhatsApp messenger

*Constant component*: All participants will receive pamphlets emphasizing the importance of primary teeth, correct feeding habits, recommended sugar intake, and age-appropriate oral hygiene practices. Toothpaste samples will be distributed as tokens of appreciation. This component aims to provide standard basic oral health education and foster engagement and trust.

*Motivational interviewing*: A single 30–45-min MI session will be offered by one researcher (MI) experienced in motivational interviewing for promoting oral health care. This component will target the perceived control construct of the TPB. The researcher providing the MI will receive training using the MI network of trainers’ resources [24]. The MI session includes establishing rapport and encouraging participants to talk about their dental habits and what they want for their children to motivate them to accept changes in behaviors. Options and strategies for improving dental health will be discussed. After the session, parents will be contacted by phone every 2 months, for 6 months, to reinforce commitment to the new behavior and provide support. Without follow-up, new behaviors may not be maintained or even tried leading to relapse.

*Oral health promotion messages*: Teledentistry and mobile health (mHealth) are proving to be an effective tool for the promotion of oral health in various age groups [25]. mHealth components will be incorporated in the proposed study to reduce the time needed for parents to receive health education, adhere to physical distancing in the time of COVID-19, and make use of the popularity of these tools among the younger generations. A series of unique messages targeting the subjective norms construct of the TBP will be developed by the researchers (MI, MET, and WE) and sent to the parents using the WhatsApp Messenger [26]. The messages are based on previous studies [23, 27] and are modified to fit the proposed study. The validity, acceptability, preferred frequency, and timing of messages will be determined in the feasibility pilot study of the preparation phase (Appendix 1). Based on the frequency, the number of messages will be determined so that they are spread over the study period.

The following are examples of the messages:

“{name}, this is {dentist} from {clinic}. Did you know that brushing your children’s teeth twice daily using a toothbrush and toothpaste will help them have a nicer smile in front of their friends and family?”

“{name}, this is {dentist} from {clinic}. Ensure smaller dental bills for your family by brushing your child’s teeth twice daily using a toothbrush and toothpaste.”

“{name}, this is {dentist} from {clinic}. Be a role model for your children and show them how to brush their teeth twice daily using a toothbrush and toothpaste”.

*Storytelling videos*: Via WhatsApp messenger, the parents will receive another mHealth component: a series of 1-min videos targeting the attitude construct of the TPB and prepared by the investigators (MI, MET, and WE). The videos have a scenario narrating the experiences of parents whose children suffered from ECC, emphasizing the positive effect of tooth brushing and fluoridated toothpaste in controlling ECC, and enhancing children’s oral hygiene. The preferred frequency and timing of the videos will also be assessed during the preparation phase (Appendix 1) and based on the frequency; the number of videos will be determined.

##### c. Feasibility pilot study

The feasibility pilot study assesses the acceptability and features of MI, OHPM, and ST videos. The feasibility study is not planned to be powered. Ten to 16 parents will be recruited from the clinic of the Department of Pediatric Dentistry and Dental Public Health, Faculty of Dentistry, Alexandria University, Alexandria, Egypt, until information saturation occurs [28]. Each parent will receive and provide feedback on the MI session and phone calls, the OHPM, and the ST videos.

*Inclusion criteria for parents in the feasibility pilot study:*

a- Has children between 2 and < 5 years of age
b- Has children with dental plaque index scores ≥ 2 or visible plaque accumulation on maxillary anterior teeth
c- Literate and owns a mobile phone with WhatsApp messenger application or where WhatsApp messenger can be installed
d- Willing to participate in the study

*Exclusion criteria:*

a- Parents of children with a definitely negative behavior (Frankel’s scale rating 4) [29]
b- Parents of physically disabled or medically compromised children
c- Parents of children who need emergency dental treatment

The parent will sign an informed consent form and receive the 3 components. The acceptability of the 3 components will be evaluated in a semi-structured interview using the framework of acceptability [30] assessing: affective attitude, burden, perceived effectiveness, ethicality, intervention coherence, and opportunity costs (Appendix 1). They will also be asked about the preferred frequency and timing of the messages and videos.

##### d. Setting the optimization objective

The optimization objective is to develop an intervention that fits within the time a parent is willing to spend receiving health education to support him/her to brush their child’s teeth. This objective may be more relevant in the mother and child health centers where the optimized intervention is planned to be provided. In this setting, parents receive ante- and post-natal care including health education. These services are provided free of charge by salaried providers, and thus, financial constraints and provider time may not be appropriate constraining factors. The key constraint [19] in the present study is the time the parent is willing to spend receiving the components. This time should cover commuting to and from the clinic to receive MI, the time of the phone call reminders for MI, the time to read the WhatsApp messages, and that spent watching the videos. It will be assessed using a questionnaire to the parents in the feasibility pilot study (Appendix 1).

#### II. Optimization phase

##### a. Factorial trial design

A factorial trial will be used to compare the levels of the three intervention components: MI, OHPM, and ST videos with the features identified during the preparation phase. Factorial designs are preferred for comparing the three components for two reasons. Firstly, factorial experiments separate component effects, enabling the estimation of the main effect and interactions between components. Secondly, factorial trials are efficient compared to alternative designs because they require fewer participants to achieve the same statistical power. Conducting three individual trials will require three times as many participants as the factorial trial.

*Study setting*: Outpatient clinics of the Department of Pediatric Dentistry and Dental Public Health, Faculty of Dentistry, Alexandria University, Alexandria, Egypt. This outpatient clinic is part of the largest public hospital in the city and is affiliated with Alexandria University. It receives hundreds of patients daily and is a suitable setting for patient recruitment.

*Eligibility criteria*: The inclusion and exclusion criteria are the same as those of the feasibility pilot study.

*Intervention components*: Each of the three components (MI, OHPM, and ST) has two levels: yes (the intervention is applied) and no (the intervention is not applied) as shown in Table 2. Participants will be divided into subgroups, each receiving one of the eight experimental conditions.

**Table 2:** Factorial trial design in the optimization phase with 8 experimental conditions

Experimental condition	MI	OHPM	ST
1	No	No	No
2	No	No	Yes
3	No	Yes	No
4	No	Yes	Yes
5	Yes	No	No
6	Yes	No	Yes
7	Yes	Yes	No
8	Yes	Yes	Yes

*Outcome measures*: The outcome assessment uses validated methods and assessment tools including a clinical examination and a questionnaire. Therefore, these tools and methods will not be further assessed in the pilot feasibility study.

1. Primary outcome: Reduction in the dental plaque index scores of children of participating parents after 3 and 6 months [31] will be measured using the modified plaque index (PlI) of Silness and Löe [32] by another researcher than the one providing the intervention (NA). The World Health Organization (WHO) community periodontal index of treatment needs (CPITN) probe and a disposable dental mirror will be used to assess plaque on 6 index teeth (upper right 2nd primary molar, upper primary right lateral incisor, upper left 1st primary molar, lower left 2nd primary molar, lower left primary lateral incisor, and lower right 1st primary molar) at baseline and after 3 and 6 months. The four surfaces of each tooth will be scored from 0 to 3 and the scores will be averaged per tooth. The scores of the six teeth will be averaged to obtain the mean PlI of the child. The outcome measure is the percent reduction in dental plaque scores calculated as: [(plaque score after 3 or 6 months − plaque score at baseline)/ plaque score at baseline] × 100. The primary outcome measure is reduction in plaque index scores after 6 months.

2. Secondary outcome: Parent-reported frequency of brushing the child’s teeth using fluoridated toothpaste will be assessed by the Arabic version of the WHO questionnaire [33] at baseline, after 3, and 6 months. The questionnaire assesses the frequency of toothbrushing on a 7-point scale ranging from never to twice or more a day. The outcome measure is the change in frequency from baseline to 3 or 6 months categorized as increased (changing from lower to higher frequency), remained the same (reporting the same frequency) and decreased (changing from higher to lower frequency). (Appendix 2)

3. Moderators such as child’s age and sex, the number of children in the family, parents’ age, oral hygiene practices, and education level will be assessed by the questionnaire.

**Fig. 2:**
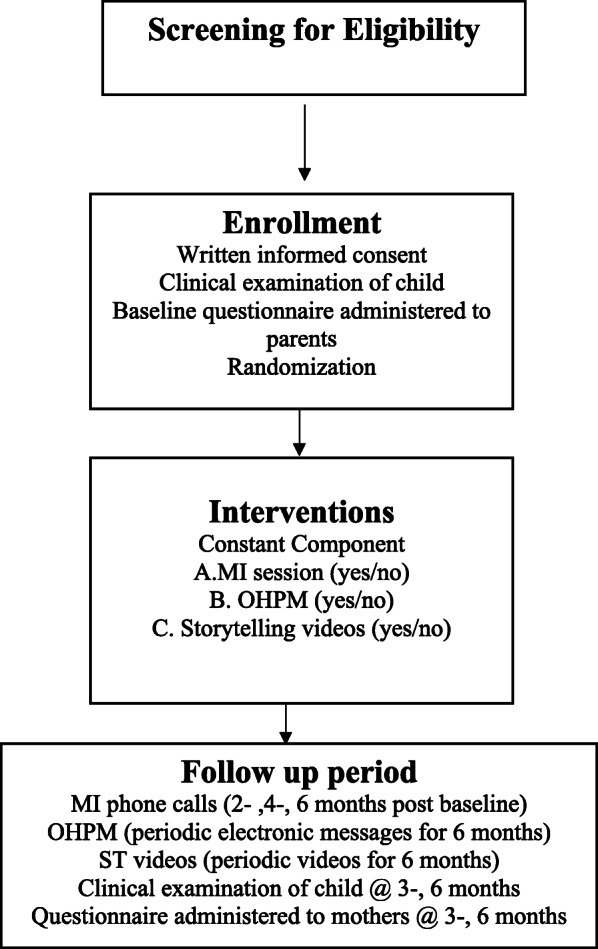
Flow chart of participants throughout the factorial trial of the optimization phase

*Participant timeline*: Participant timeline is shown in Fig. 2.

*Sample size calculation*: The study is powered to detect at least a medium effect size measured by partial eta squared (*ƞ*^2^) = 0.06; the effect size measure for factorial ANOVA [34]. In G* Power 3.0.10 [35], we specified *f*= 0. 0.2526456 based on the required *ƞ*^2^, alpha error= 5% and study power= 80%. For the 8 conditions in Table 2, we used numerator df= (2 levels in MI- 1) X (2 levels in OHPM- 1) X (2 levels in ST videos- 1) = 1 X 1 X 1= 1. A total sample size of 126 will allow the estimation of main effects and interactions using linear regression analysis. This will be rounded to 160 corresponding to 20 parents in each of the 8 experimental conditions.

*Randomization and allocation concealment*: Parents will be assessed for eligibility and randomly allocated by the Ralloc Stata Software [36] to the 8 experimental conditions (Table 2) using a permuted block of size 16, to be able to balance the sample size across the randomization groups. Each block will be a list of 8 randomly ordered conditions, and participants will be randomized to the condition listed in the permuted block. Once all allocations in a block have been used, the next permuted block will be utilized. The randomization schedule will be kept in opaque, sealed envelopes, and the envelopes will be arranged sequentially by a dental assistant who will not be involved in the study. Each envelope will be opened after completing the child’s oral examination at baseline and the experimental condition to which the parent is allocated will be administered. Parents’ enrollment, random sequence generation, and allocation to experimental conditions will be done by an independent researcher (RY) who will be different from the researcher implementing the experimental conditions and assessing the outcomes. The researcher also clinically assessing plaque accumulation (primary outcome) (NA) and the researchers involved in data analysis (MI, MT) will be blinded to the assignment to interventions. At the end of the 6-month period of the study, all children in need of any dental treatment will be referred to a pedodontist in the outpatient clinic. Participants who will be lost to follow-up or who choose to withdraw from the study will be noted and their reasons for doing so as well as the group to which they were allocated will be recorded and reported in the study results*.* All attempts will be made to collect data for the study outcome measures for participants who discontinue or deviate from the interventions.

##### b. Selecting the most efficacious components fitting the optimization objective

The combination of components making up the optimized intervention will be selected according to the following procedure [37]:

- The three components are labeled *A* to *C*.
- The main effect of component A will be defined as the difference between the yes level and the no level of that component, averaged across all the levels of components *B*–*C.* This will be calculated by subtracting the mean response in conditions 1–4 in Table 2 from the mean response in conditions 5–8.
- A two-way interaction involving components *A* and *B* will occur if the effect of *A* at the “Yes” level of *B* is different from the effect of *A* at the “No” level of *B*, averaged across all the levels of *C* and *D.*
- Regression coefficient estimates will be produced by conducting a factorial ANOVA using effect coding (−1,1).
- If a component has a main effect with at least a moderate effect size, the “yes” level will be chosen for inclusion in the intervention. If the component does not achieve a moderate effect size or has an effect in the wrong direction, such as increasing the plaque index, the “no” level will be selected. This allows the inclusion of efficacious components and the elimination of components which have no use.
- An initially selected component may be deselected if it interacts with another component which undermines its effect, or a component not initially selected may be selected if it interacts with another component to enhance its effect.
- The combination of components which fits within the constraint of the optimization objective will be selected for inclusion in the optimized intervention package.

### Data management and statistical analysis

IBM SPSS Statistics for Windows (Version 25.0. Armonk, NY: IBM Corp) [38] will be used for data analysis. A linear regression model will be used to assess the effect of the components on percent reduction in plaque index score adjusting for the following confounders: parent’s sex, age, education, occupation, brushing using fluoridated toothpaste, number of children in the family, child’s age, and sex. The percent reduction in plaque index scores will be assessed for the subgroups included in the adjusted analysis. All participants randomized into the 8 conditions will be included in the analysis following an intention to treat analysis including those receiving a co-intervention, those with contaminated intervention, and those who do not receive the interventions in the oral health promotion messages and the story telling videos. For participants who drop from the study and have no outcome measures data, multiple imputation will be used to derive the missing data values. Significance will be set at the 5% level.

### Data monitoring

An interim analysis of the data collected at the 3 months’ follow-up will be done to assess progress in the outcome measures in response to the delivery of the interventions. Due to the non-invasive nature of the interventions, no stopping rules will be made to terminate the study and emphasis will be made that participants are free to withdraw from the study if the interventions become unacceptable. No Data Monitoring Committee is available in the researcher’s institution and therefore no external body outside the study team will be monitoring the data.

### Harms

Participants will be contacted by phone every 2 months, for the duration of the trial to assess reported adverse events or any other unintended effects. This will also be assessed at the 3 months’ follow-up appointment.

### Auditing

No auditing is planned or expected.

### Ethics approval and consent to participate

The study received approval from the Ethics Committee of the Faculty of Dentistry, Alexandria University. IRB No: 00010556-IORG: 0008839. A signed informed consent will be obtained by the principal investigator (MI) from each parent. The informed consent that will be signed by the parents will encompass the data to be collected and its intended analysis and use for the study purpose. No additional studies are planned, and no biological samples are to be collected because of the focus of the study on parents’ behavior and its impact on plaque accumulation in children. The voluntary nature of the study will also be emphasized in the consent form, and the parents will be informed of their right to withdraw from the trial anytime, without incurring any penalties. In addition, no advice will be given to the participants to avoid receiving or seeking any health education content.

### Biological samples

No biological samples will be collected in this trial and therefore, no plans are made for their collection, evaluation, or storage.

### Trial status

This is the original version of the protocol, issued on the 9th of June 2021. The recruitment is planned to start on the 20th of August 2021 and end approximately by February 2022. Any changes or protocol amendments will be made to the study record available on clinical trials.gov.

### Confidentiality

Identification numbers will be used to assure participant confidentiality during data analysis. All data will be collected using a secure, web-based, and password-protected database.

### Ancillary and post-trial care

There are no harms or expected adverse outcomes from the interventions to be used in this trial. This is because the scope of the study is the delivery of health education using different methods.

### Dissemination policy

The results of the study will be disseminated through international peer-reviewed journals and conferences. There is no intention to use the services of professional writers. All authors of the final manuscript will fulfill the authorship criteria as recommended by the International Committee of Medical Journal Editors. The full protocol is also provided as a supplement and participant-level dataset and statistical code will be made available on reasonable request from the principal investigator.

## Discussion

This study adopts an innovative research method to develop an efficient and scalable behavior modification intervention based on participants’ acceptance and preferences using components that are likely to accommodate the conditions during the time of the pandemic. The literature about young children’s oral hygiene provides little guidance on optimal intervention designs and existing evidence describes components which vary in effectiveness. Little is known from the literature about the underlying theoretical framework, form, and dose of interventions to elicit effective behavior change. Previous studies [39] used packages of multiple interventions but none of them assessed the efficacy of individual components. Our study is considered the first to apply the MOST framework in the field of dentistry. Through MOST, we hope to isolate the components then assemble an efficacious standalone intervention package that fits the optimization criterion.

## Supplementary Information

**Additional file 1:** Appendix 1 Acceptability of Proposed Intervention Components. Appendix 2 World Health Organization Oral Health Assessment Questionnaire.

## Data Availability

The final dataset of the proposed study will be available upon reasonable request from the corresponding author.

## Abbreviations

ECC: Early childhood caries
MOST: Multi-phase optimization strategy
RCTs: Randomized clinical trials
TPB: Theory of planned behavior
MI: Motivational interviewing
OHPM: Oral health promotion messages
ST: Storytelling
