# Supplementary material for: Using mHealth to promote parents’ brushing of preschool children’s teeth: a protocol for a randomized factorial trial using the Multi-phase Optimization Strategy (MOST)
Source: Trials. 2022 Jan 6;23:17. doi: 10.1186/s13063-021-05931-0 (PMC8733768; doi:10.1186/s13063-021-05931-0)
Supplement: Supplementary file 1 — Additional file 1: Appendix 1 Acceptability of Proposed Intervention Components. Appendix 2 World Health Organization Oral Health Assessment Questionnaire. [file 13063_2021_5931_MOESM1_ESM.docx]

**Appendix 1**

**Acceptability of Proposed Intervention Components**

**Section 1: Sociodemographic Characteristics**

1. **Parent/ Child ID:** ...............
2. **Parents’ name:** .................
3. **Parents’ age (In years):** .......................
4. **What level of education have you completed?**

- No formal schooling/ less than primary school
- Primary school completed
- High school completed
- College/university completed
- Postgraduate degree

**5. Occupation:**

- Does not work / Housewife
- Unskilled manual worker (porters - day laborers - messengers)
- Skilled manual worker (owners of crafts and industrial industries)
- Traders and businessmen
- Government employees and administrators
- Professionals (doctors - university professors - lawyers - officers)

**Section 2: Perceived and Experienced Acceptability**

**On a scale of 1-10, please indicate to what extent you agree with the following statements**

1. I enjoyed receiving the intervention

1 2 3 4 5 6 7 8 9 10

1. The intervention required too much effort

1 2 3 4 5 6 7 8 9 10

1. The content of the intervention was easy to understand and follow

1 2 3 4 5 6 7 8 9 10

1. This intervention is not ethical or in line with my personal values

1 2 3 4 5 6 7 8 9 10

1. Implementing the intervention would cause me trouble or problems

1 2 3 4 5 6 7 8 9 10

1. I would have to give up something else important because of the intervention

1 2 3 4 5 6 7 8 9 10

1. The interventions would be effective in motivating other parents to brush their children’s teeth

1 2 3 4 5 6 7 8 9 10

**Section 3: Preferred frequency and timing of receiving the components**

1. **I prefer to receive the messages/ videos:**

- 2 times/ week
- 1 times/ week
- 3 times/ month
- 2 times/ month
- 1 times/ month
- Less than that (please define): ……...

1. **I prefer if the messages/ videos are sent:**

- After 8am to 2pm
- After 2pm to 8pm
- After 8pm to 2am
- After 2am to 8am

**Section 4: Specifying an Optimization objective**

**What is the maximum total time are you willing to spend receiving education on how to brush your child’s teeth?**

- 0-15 min
- >15-30 min
- >30-45 min
- >45-60 min
- >60-75 min

**Appendix 2**

**World Health Organization Oral Health Assessment Questionnaire**

1. Parent / Child ID: ......
2. Date: .......
3. Child sex: Male / Female
4. Age: ......
5. How many children do you have? .....
6. How old is your child? .......
7. How would you describe the state of your teeth and gums?

- Excellent
- Very good
- Good
- Average
- Poor
- Very poor
- Don’t know

1. How would you describe the state of your child’s teeth and gums?

- Excellent
- Very good
- Good
- Average
- Poor
- Very poor
- Don’t know

1. How often do you clean your teeth?

- Never
- Once a month
- 2–3 times a month
- Once a week
- 2–6 times a week
- Once a day
- Twice or more a day

1. How often do you clean your child’s teeth?

- Never
- Once a month
- 2–3 times a month
- Once a week
- 2–6 times a week
- Once a day
- Twice or more a day

1. Do you use any of the following to clean your teeth? (Read each item)

- Toothbrush
- Wooden toothpicks
- Plastic toothpicks
- Thread (dental floss)
- Charcoal
- Chewstick/miswak
- Other
- Please specify

1. Do you use any of the following to clean your child’s teeth?

- Toothbrush
- Wooden toothpicks
- Plastic toothpicks
- Thread (dental floss)
- Charcoal
- Chewstick/miswak
- Other
- Please specify

1. Do you use toothpaste to clean your teeth?

- Yes
- No

1. Do you use a toothpaste that contains fluoride?

- Yes
- No
- Don’t know

1. Do you use toothpaste to clean your child’s teeth?

- Yes
- No

1. Do you use a toothpaste that contains fluoride for your child?

- Yes
- No
- Don’t know
